# Early Noninvasive Metabolic Biomarkers of Mutant IDH Inhibition in Glioma

**DOI:** 10.3390/metabo11020109

**Published:** 2021-02-13

**Authors:** Marina Radoul, Donghyun Hong, Anne Marie Gillespie, Chloé Najac, Pavithra Viswanath, Russell O. Pieper, Joseph F. Costello, Hema Artee Luchman, Sabrina M. Ronen

**Affiliations:** 1 Department of Radiology and Biomedical Imaging, University of California, San Francisco, CA 94158, USA; marina.radoul@ucsf.edu (M.R.); donghyun.hong@ucsf.edu (D.H.); annemarie.gillespie@ucsf.edu (A.M.G.); c.f.najac@lumc.nl (C.N.); pavithra.viswanath@ucsf.edu (P.V.); 2 Department of Neurological Surgery, Helen Diller Research Center, University of California, San Francisco, CA 94158, USA; Russ.pieper@ucsf.edu (R.O.P.); joseph.costello@ucsf.edu (J.F.C.); 3 Brain Tumor Research Center, University of California, San Francisco, CA 94158, USA; 4 Arnie Charbonneau Cancer Institute and Hotchkiss Brain Institute, University of Calgary, Calgary, AB T2N 4N1, Canada; aluchman@ucalgary.ca

**Keywords:** noninvasive metabolic biomarkers, mutant IDH inhibitor, glioma, ^1^H-MRS

## Abstract

Approximately 80% of low-grade glioma (LGGs) harbor mutant isocitrate dehydrogenase 1/2 (IDH1/2) driver mutations leading to accumulation of the oncometabolite 2-hydroxyglutarate (2-HG). Thus, inhibition of mutant IDH is considered a potential therapeutic target. Several mutant IDH inhibitors are currently in clinical trials, including AG-881 and BAY-1436032. However, to date, early detection of response remains a challenge. In this study we used high resolution ^1^H magnetic resonance spectroscopy (^1^H-MRS) to identify early noninvasive MR (Magnetic Resonance)-detectable metabolic biomarkers of response to mutant IDH inhibition. In vivo ^1^H-MRS was performed on mice orthotopically-implanted with either genetically engineered (U87IDHmut) or patient-derived (BT257 and SF10417) mutant IDH1 cells. Treatment with either AG-881 or BAY-1436032 induced a significant reduction in 2-HG. Moreover, both inhibitors led to a significant early and sustained increase in glutamate and the sum of glutamate and glutamine (GLX) in all three models. A transient early increase in N-acetylaspartate (NAA) was also observed. Importantly, all models demonstrated enhanced animal survival following both treatments and the metabolic alterations were observed prior to any detectable differences in tumor volume between control and treated tumors. Our study therefore identifies potential translatable early metabolic biomarkers of drug delivery, mutant IDH inhibition and glioma response to treatment with emerging clinically relevant therapies.

## 1. Introduction

Diffuse infiltrative low-grade gliomas (LGGs) are characterized as grade II or III astrocytoma and oligodendroglioma based on histopathological features and genotype including the status of the isocitrate dehydrogenase (IDH) mutation, 1p/19q codeletion and ATRX [1,2,3]. LGGs typically affect young or middle-aged adults [4]. Diagnosis of LGGs is typically based on symptoms, the most notable of which is seizures, and is confirmed via imaging of non- or mildly contrast enhancing lesions using magnetic resonance imaging (MRI) [5,6]. The previous treatment approach of *wait-and-see* has currently been replaced by maximum safe surgical resection and has been shown to enhance the survival of LGG patients [7]. Additional treatment, including radiotherapy, chemotherapy or combined treatments, is decided on a case-by-case basis [5,8]. However, one of the challenges in the management of LGGs is their highly infiltrative nature, which limits full surgical resection and leaves behind neoplastic cells within normal-appearing brain tissue [9]. As a result, in most cases, tumor recurrence and malignant transformation to a higher grade will occur leading to progressive disabilities and shortened survival [10].

Over 80% of LGGs harbor somatic mutations in IDH1 or IDH2 leading to a neomorphic enzyme activity that catalyzes the production of the oncometabolite 2-hydroxyglutarate (2-HG) from α-ketoglutarate (α-KG) [11,12,13]. 2-HG drives tumorigenesis through competitive inhibition of α-KG-dependent dioxygenases, including histone demethylases and DNA hydroxylases [12,14]. The role of the IDH mutation as an oncogenic driver, and its recognition in 2016 by the World Health Organization as a crucial genetic characteristic of LGGs, have stimulated extensive exploration of therapies that could inhibit 2-HG production and potentially reverse its oncogenic effects [2,11,15,16,17]. In particular, several promising mutant IDH inhibitors including AG-120, IDH305, AG-881, BAY-1436032, AG-221, and FT-2120 have been developed and are currently in ongoing clinical trials for gliomas either alone or in combination with chemotherapy (NCT02073994, NCT02381886, NCT03343197, NCT02481154, NCT04164901, NCT02746081, NCT02273739, NCT03684811) [18].

Clinical evaluation of glioma growth rate, malignant transformation and monitoring of response to treatment, as well as surgical planning and targeting of radiation therapy, are most commonly based on noninvasive imaging using a range of MRI approaches [9]. Current standardized recommendations for brain tumor MRI primarily include T2-weighted, FLAIR (Fluid Attenuated Inversion Recovery), and pre- and postcontrast T1-weighted imaging [19]. Additionally, advanced MRI techniques including diffusion-weighted imaging (DWI) and susceptibility-weighted imaging (SWI) are also performed for brain tumor evaluation [19]. Finally, functional MRI (fMRI) can be used to identify areas of neurological function during preoperative evaluation and surgical planning [19].

Another MR-based imaging method is MR Spectroscopy (MRS), which in the clinic has most often been reserved as a complementary technique for tumor diagnostics and grading [20,21,22,23]. Importantly, MRS noninvasively detects metabolite levels [24]. As a result, since the discovery of the IDH mutation, this method has the potential to play an important role in noninvasive detection of 2-HG and thus tumor characterization. 2-HG is sometimes present in relatively low levels and its signals overlap with other metabolites, but this challenge has been addressed by development of several advanced data acquisition methods that allow for monitoring of 2-HG both preclinically and clinically [23,25,26,27,28,29,30,31,32]. Indeed, ^1^H-MRS has been used to monitor the effects of emerging mutant IDH1/2 inhibitors preclinically and in one early clinical study with IDH305 [33,34]. The use of MRS is particularly important because clinical studies to date with three mutant IDH inhibitors, AG-881, IDH305, and AG-120, have all shown that treatment is associated with inhibition of tumor growth and potentially longer patient survival, but no detectable tumor shrinkage has been reported [33,35,36]. Thus, identification of noninvasive metabolic biomarkers that can probe metabolic changes during treatment and assess early response are critically needed to improve timely and individually tailored glioma patient management.

Our previous studies in cells have identified the MRS-detectable metabolic alterations characteristic of glioma harboring the IDH1 mutation when compared to wild type IDH1 [37]. We also showed that treatment of cells with the IDH inhibitors AG-120 and AG-881 resulted in a partial reversal of the metabolic alterations caused by the mutation [34]. Most notably, we observed that in addition to the expected ^1^H-MRS detectable drop in 2-HG, there is a significant increase in glutamate (Glu) following mutant IDH inhibition. The goal of this study was to expand upon our previous work in cells, and to assess the impact of mutant IDH inhibitors on the ^1^H-MRS spectrum of tumors in vivo particularly metabolic changes other than 2-HG. We also wanted to determine whether MRS-detectable metabolic changes could serve to predict response to therapy as determined by enhanced animal survival. To this end, we investigated three orthotopic mutant IDH1 glioma mouse models and studied the effects of two brain penetrant mutant IDH inhibitors, AG-881 and BAY-1436032, that are currently in clinical trials for glioma patients. Our findings demonstrate that in addition to a drop in 2-HG, increased Glu and the sum of Glu and glutamine (GLX) are early and sustained metabolic alterations that are associated with enhanced animal survival. Our findings thus identify clinically translatable metabolic biomarkers that could assist in monitoring response to therapy with emerging mutant IDH inhibitors.

## 2. Results

### 2.1. In Vivo ^1^H-MRS Studies Using U87IDHmut Genetically Engineered Model

Previous in vitro studies probed the effect of mutant IDH inhibitor AG-881 in U87IDHmut cells that were genetically engineered to express mutant IDH1 [34]. Therefore, prior to investigating patient-derived (BT257 and SF10417) mutant IDH1 glioma models, we first performed a small-scale study investigating the effect of AG-881 treatment on mice with orthotopic U87IDHmut tumors. Additionally, we wanted to confirm that our findings were not specific to one inhibitor and therefore also investigated a second clinically relevant IDH inhibitor namely the pan-mutant IDH1 inhibitor, BAY-1436032.

**Figure 1:**
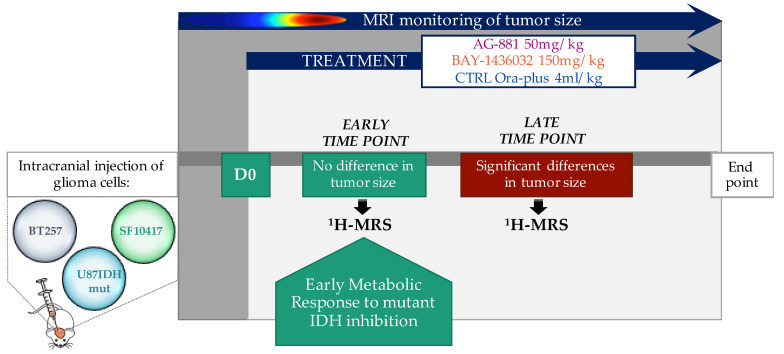
Schematic representation of in vivo ^1^H-MRS study design for orthotopic glioma-bearing mouse models treated with mutant IDH inhibitors.

**Figure 2:**
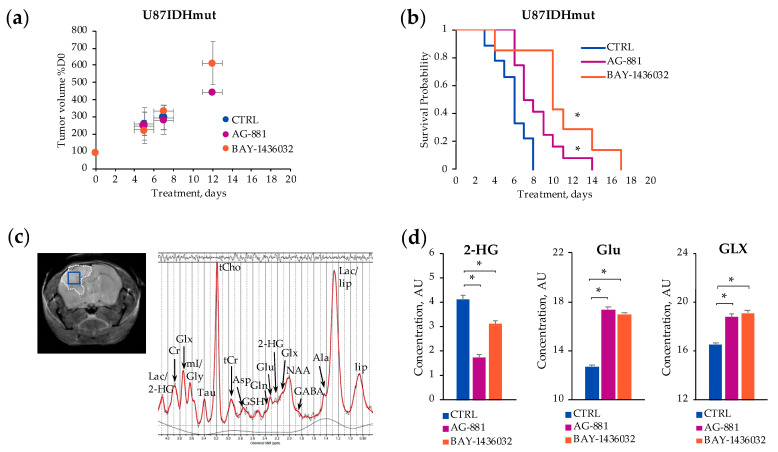
Temporal evolution of average U87IDHmut tumor volume as a percentage of D0 (**a**). Kaplan–Meier survival plot of U87IDHmut-bearing mice (* *p*-value ≤ 0.05) (**b**). A representative axial T_2_-weighted image of control U87IDHmut-bearing mouse and corresponding in vivo ^1^H-MRS spectrum acquired from the voxel marked in blue on the image (**c**). Quantification of 2-HG, Glu and GLX concentrations acquired from the voxel placed in control, AG-881- and BAY-1436032-treated tumors at D5 ± 1 (* *p*-value < 0.003) (**d**).

Figure 1 illustrates the in vivo ^1^H^-^MRS study design. U87IDHmut tumor-bearing mice were monitored regularly by MRI until tumors reached a volume of ~8 mm^3^ at 27 ± 4 days post-inoculation, at which time single voxel ^1^H-MRS was performed and treatment was initiated (D0). Figure 2a illustrates the evolution of average U87IDHmut tumor volume in control, AG-881 and BAY-1436032 treated groups. For the duration of our study, no significant changes in tumor volume could be detected following treatment with either of the mutant IDH inhibitors when compared to controls. Nonetheless, the overall survival of U87IDHmut tumor-bearing mice was significantly enhanced in both treatments. Following AG-881 treatment the median survival was 7.5 days (*Χ*^2^-value = 26.9) and following BAY-1436032 treatment median survival was 10 days (*Χ*^2^-value = 54.7) compared to 6-day median survival of controls (Figure 2b).

We therefore focused only on the spectra acquired at D5 ± 1 of treatment, when tumor volume changes, as a percentage of D0, were 205 ± 106% (*n* = 10, *p*-value = 0.799) in the AG-881 treated group and 225 ± 58% (*n* = 6, *p*-value = 0.318) in the BAY-1436032 treated group compared to 261 ± 67% in the control group (*n* = 7) (Figure 2a). A typical in vivo ^1^H-MRS spectrum acquired from a 2 × 2 × 2 mm^3^ voxel placed inside the tumor region is shown in Figure 2c. Figure 2d illustrates the quantification of metabolites previously shown to be altered by AG-881 treatment in cell studies, namely 2-HG and Glu. We also illustrate the levels of GLX, which represents Glu plus glutamine (Gln) and is typically easier to detect in vivo (Figure 2d). Additional metabolic alterations are summarized in Table 1. As expected, the in vivo ^1^H-MRS experiments revealed a significant decrease in the concentration of 2-HG following treatment with both inhibitors (decrease by 58% in AG-881-treated and by 25% in BAY-1436032-treated) when compared to controls consistent with drug delivery and action via mutant IDH inhibition by D5 ± 1 (Figure 2d). Additionally, treatment with both inhibitors led to a significant increase in Glu (by 36% in AG-881-treated and by 34% in BAY-1436032-treated) compared to controls. GLX levels also increased significantly in response to both AG-881 (increase by 14%) and BAY-1436032 (increase by 16%) when compared to controls (Figure 2d). Other detected differences in metabolites levels are shown in Table 1.

### 2.2. In Vivo ^1^H-MRS Studies Using Patient-Derived BT257 and SF10417 Glioma Models

**Figure 3:**
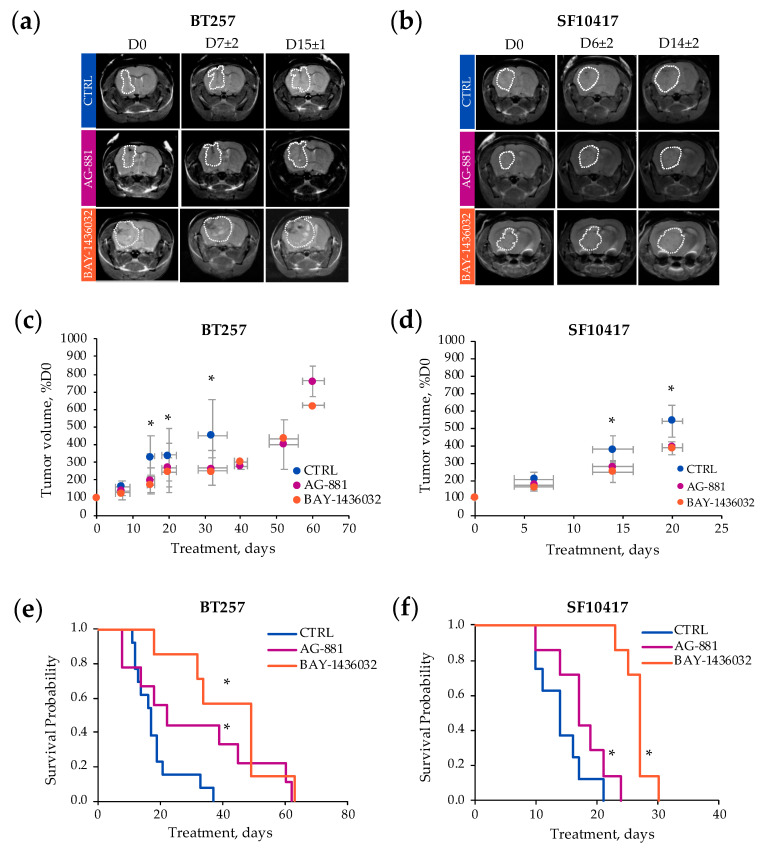
Representative axial T2-weighted images of control (top line, blue), AG-881 (middle line purple) and BAY-1436032 (bottom line orange)-treated BT257 tumor-bearing mice at D0, D7 ± 2 and D15 ± 1 (**a**) and SF10417 tumor-bearing mice at D0, D6 ± 2 and D14 ± 2 (**b**). Temporal evolution of average tumor volume (percentage of D0) for the BT257 (**c**) and the SF10417 (**d**) models (control in blue, AG-881 in purple, BAY-146032 in orange). Kaplan-Meier survival plot of BT257 (**e**) and SF10417 (**f**) tumor-bearing mice treated with either AG-881 or BAY-1436032 when compared to controls. * *p*-value ≤ 0.05.

Next, we wanted to assess the generality of our observations by investigating the patient-derived BT257 and SF10417 glioma models. Orthotopically inoculated BT257 and SF10417 cells formed ~8 mm^3^ MR-detectable homogeneous low T2-weighted contrast tumors at 94 ± 25 and 76 ± 21 days, respectively. Figure 3 demonstrates T2-weighted images of control, AG-881 and BAY-1436032-treated mice at D0 prior to treatment, D7 ± 2 and D15 ± 1 of treatment for the BT257 model, and D6 ± 2 and D14 ± 2 of treatment for the SF10417 model (Figure 3a,b). No significant differences in average tumor volume were observed in response to AG-881 in the BT257 model at D7 ± 2 (controls: 166 ± 34%, *n* = 8; AG-881-treated: 141 ± 27%, *n* = 11, *p*-value = 0.107) and in the SF10417 model at D6 ± 2 (controls: 200 ± 36%, *n* = 5; AG-881-treated: 177 ± 35%, *n* = 7, *p*-value = 0.298). However, differences in tumor volume did become significant by D15 ± 1 in the BT257 model (controls: 335 ± 117%, *n* = 9; AG-881-treated: 202 ± 68%, *n* = 6, *p*-value = 0.016) and by D14 ± 2 in the SF10417 model (controls: 381 ± 78%, *n* = 5; AG-881-treated: 280 ± 41%, *n* = 5, *p*-value = 0.042) (Figure 3c,d). Similarly, no significant differences in average tumor volume were observed at the early time point of treatment with BAY-1436032 in the BT257 model at D7 ± 2 (BAY-1436032-treated: 132 ± 28%, *n* = 5, *p*-value = 0.142) and in the SF10417 model at D6 ± 2 (BAY-1436032-treated: 166 ± 17%, *n* = 7, *p*-value = 0.102). But we detected a significant slowdown in tumor growth in response to BAY-1436032 treatment by D15 ± 1 in the BT257 model (BAY-1436032-treated: 174 ± 53%, *n* = 5, *p*-value = 0.004) and by D14 ± 2 in the SF10417 model (BAY-1436032-treated: 252 ± 60%, *n* = 6, *p*-value = 0.018) when compared to controls (Figure 3c,d).

The inhibition of tumor growth following treatment with both compounds also significantly improved the survival of both BT257 and SF10417-tumor bearing mice compared to controls. The median survival of BT257 and SF10417 control mice was 16 and 15 days, respectively. Following AG-881 treatment the median survival of mice bearing BT257 tumors increased to 30.5 days (*Χ*^2^-value = 49.6), whereas the median survival of mice bearing SF10417 tumors increased to 18 days (*Χ*^2^-value = 59.5). Following BAY-1436032 treatment the median survival of mice bearing BT257 tumors increased to 34 days (*Χ*^2^-value = 120.6) and the median survival of mice bearing SF10417 tumors increased to 26 days (*Χ*^2^-value = 118.8) (Figure 3e,f).

**Figure 4:**
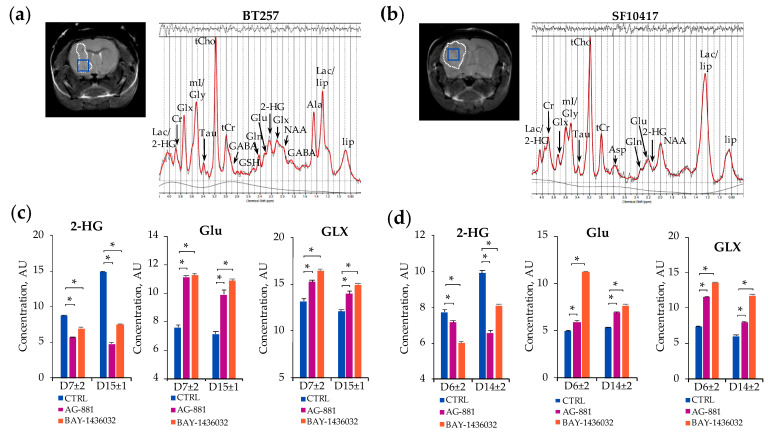
Representative axial T_2_-weighted images of control BT257 (**a**) and SF10417 (**b**) tumor-bearing mice and corresponding in vivo ^1^H-MRS spectra acquired from 2 × 2 × 2 mm^3^ voxel marked inside the tumor region in blue (**a**,**b**). Quantification of 2-HG, Glu and GLX metabolites in control, AG-881- and BAY-1436032-treated tumors at D7 ± 2 and D15 ± 1 in the BT257 (**c**), and at D6 ± 2 and D14 ± 2 in the SF10417 models (* *p*-value < 0.003) (**d**).

Based on the temporal evolution of average tumor volume described above, we next designed the spectroscopic study such that we investigated the in vivo ^1^H-MRS metabolic profile of each model in response to treatment at two time points: the early time point, D7 ± 2 for BT257 and D6 ± 2 for SF10417, prior to differences in tumor volume, and the late time point D15 ± 1 for the BT257 model and D14 ± 2 for the SF10417 model, when significant differences in tumor volume were MR-detectable (Figure 1 and Figure 3). Figure 4 illustrates typical T2-weighted anatomical images and corresponding ^1^H-MRS spectra acquired from the voxel placed inside the tumor region of the BT257 and SF10417 glioma models (Figure 4a,b).

As expected, at the early time point prior to any significant differences in tumor volume, we detected a consistent and significant decrease in 2-HG levels in response to mutant IDH inhibition. Following treatment with AG-881, 2-HG significantly dropped in both BT257 (decrease by 35%) and SF10417 (decrease by 7%) tumors (Figure 4c,d, Table 2 and Table 3). When compared to controls, lower levels of 2-HG were also observed at the later time point when inhibition of tumor growth was detected in both BT257 (decrease by 68% in AG-881-treated) and SF10417 (decrease by 34% in AG-881-treated) models. (Table 2 and Table 3). Additionally, at the early time point and, in both models, treatment with AG-881 led to significantly elevated concentrations of Glu in the BT257 (increase by 46% in AG-881-treated) and in the SF10417 (increase by 20% in AG-881-treated) models (Table 2 and Table 3). Similarly, GLX levels were also significantly increased in both models (in BT257 increase by 15% in AG-881-treated and in SF10417 increase by 57% in AG-881-treated) (Figure 4c,d). The metabolic alterations in Glu (increase by 39% in BT257 and by 32% in SF10417 tumors) and GLX (increase by 16% in BT257 and by 32% in SF10417 tumors) were sustained and remained significant also at the later stages of AG-881 treatment when changes in tumor volume were already MR-detectable (Table 2 and Table 3). Some additional metabolic changes were also observed with AG-881 treatment (Table 2 and Table 3).

Treatment of BT257 and SF10417 tumors with BAY-1436032 resulted in the same metabolic trends as treatment with AG-881. 2-HG levels were significantly decreased in response to BAY-1436032 treatment at the early time point prior to MR-detectable differences in tumor volume in the BT257 (decrease by 20% in BAY-1436032-treated) and in the SF10417 (decrease by 23% in BAY-1436032-treated) models (Figure 4c,d, Table 2 and Table 3). Significant decrease in the levels of 2-HG was also observed at the later time point in the BT257 (decrease by 50% in BAY-1436032-treated) and in the SF10417 (decrease by 18% in BAY-1436032-treated) models compared to corresponding controls (Table 2 and Table 3). Additionally, treatment with BAY-1436032 resulted in a significant increase in the levels of Glu at the early time point in both the BT257 (increase by 48% in BAY-1436032-treated) and the SF10417 (increase by 128% in BAY-1436032-treated) models. GLX was similarly significantly increased with treatment at the early time point in both the BT257 (increase by 24% in BAY-1436032-treated) and in the SF10417 (increase by 85% in BAY-1436032-treated) models when compared to controls (Figure 4c,d, Table 2 and Table 3). These increases in Glu (increase by 52% in BT257 and by 45% in SF10417 tumors) and GLX (increase by 24% in BT257 and by 95% in SF10417 tumors) were also sustained at the later time point of BAY-1436032 treatment in both models (Table 2 and Table 3).

### 2.3. Metabolic Profile Commonly Associated with Mutant IDH1 Inhibition

**Figure 5:**
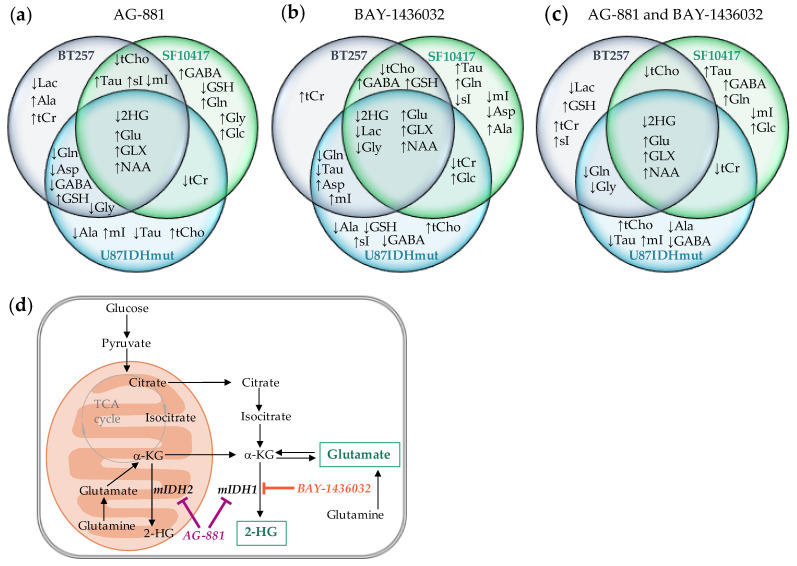
Diagrams of metabolic alterations occurring in U87IDHmut, BT257 and SF10417 models at early time points (D5 ± 1 for U87IDHmut model (blue), D7 ± 2 for BT257 model (grey) and D6 ± 2 for SF10417 model (green)) prior to MR-detectable differences in tumor volume, following treatment with mutant IDH inhibitors AG-881 (**a**) or BAY-1436032 (**b**). Diagram showing changes that occur in each model with both treatments and highlighting the metabolic changes that occur for all models with both treatments (intersection of all three circles at center) (**c**). Metabolic pathways illustrating the MR-detectable metabolites altered following mutant IDH1/2 inhibition (**d**).

Figure 5 provides a summary of the different metabolic alterations observed prior to MR-detectable changes in tumor volume in all three models following mutant IDH inhibition. In all of our models, mutant IDH inhibition with either AG-881 or BAY-1436032 led to the expected drop in 2-HG level. In addition, we observed in every case a significant increase in Glu as well as GLX.

Interestingly, we also observed a significant increase in NAA levels in all of our models at the early time point, prior to detectable changes in tumor volume. Thus, in the U87IDHmut tumors NAA increased following treatment with AG-881 by 48% and following BAY-1436032 treatment by 37% when compared to controls at D5 ± 1 (Table 1). Similarly, NAA increased in BT257 tumors following treatment with AG-881 by 33% and following treatment with BAY-1436032 by 57% when compared to controls D7 ± 2 (Table 2). Finally, in the SF10417 model NAA level increased following AG-881 and BAY-1436032 treatment by 27% and 35% respectively compared to controls D6 ± 2 (Table 3). However, the increase in NAA levels was not sustained at the later time point of treatment for either BT257 or SF10417 tumors (Table 2 and Table 3).

## 3. Discussion

The relatively recent discovery of the IDH1/2 mutations was a crucial observation that has impacted the way the World Health Organization now classifies gliomas [2]. This discovery also triggered multiple studies investigating the effect of targeted mutant IDH1/2 inhibition in gliomas and other tumor types, and a number of pan and specific mutant IDH inhibitors are in clinical trials.

AG-881 is a first in class, oral, potent inhibitor of mutant IDH1/2 [38]. It has demonstrated brain penetrance, 2-HG suppression and some disease-control in early clinical trials (NCT02481154, NCT03343197). Based on the early data this agent is now in a phase III, multicenter, randomized clinical study (NCT04164901). BAY-1436032 is an oral pan-inhibitor of mutant IDH1. It has robust activity in preclinical glioma models showing that BAY-1436032 treatment has led to significantly longer survival of astrocytoma-bearing mice [39,40]. As a result, BAY-1436032 is also now in clinical trials (NCT02746081). We therefore chose to investigate both of these inhibitors in order to confirm that any biomarkers identified in this study are not treatment specific.

Importantly, as mentioned, clinical studies of AG-881 and a second inhibitor, IDH305, have shown that although treatment inhibits tumor growth, reduces cell density, and may extend patient survival, there is no evidence of clearly detectable tumor shrinkage [33,35]. The study of yet another inhibitor, AG-120, also recently demonstrated only inhibition of tumor growth [36]. Consistent with these observations, in this study, we found that treatment of both genetically engineered and patient-derived models, with either AG-881 or BAY-1436032, led, at best, to inhibition in tumor growth (BT257 and SF10417) when compared to corresponding controls, but no tumor shrinkage could be observed. Nonetheless, overall survival was significantly longer in all three treated mutant IDH1 models in response to both AG-881 and BAY-1436032 treatments. The lack of frank tumor shrinkage and continued tumor growth, albeit at a slower rate, highlights the need for early noninvasive metabolic biomarkers of drug delivery, drug action, and response to mutant IDH inhibition using complementary noninvasive imaging methods such as ^1^H-MRS.

In this study we investigated three different mutant IDH1 tumor models. The first, U87IDHmut, served to confirm that our previous findings in U87IDHmut cells also hold in the in vivo setting. However, the U87IDHmut model is genetically engineered to express mutant IDH1 in U87 glioblastoma cells. As such, it would carry oncogenic mutations that are characteristic for glioblastoma, and any observation made in this model could be associated with its glioblastoma background. We therefore also performed more extensive studies in two patient-derived glioma models: an astrocytoma (BT257) and an oligodendroglioma (SF10417). Interestingly, we found metabolic alterations that were common to all our models, pointing to their reliable nature and their lack of dependence on other factors such as invasion, blood brain barrier, or oncogenic mutations other than IDH1.

Our study benefited from the use of a high field 14.1 T animal scanner, which allowed us to distinguish the different metabolites and probe their modulation with treatment. In the clinical setting MR scanners are typically at the lower fields of 1.5 T or 3 T. However, as mentioned above, sequences specifically optimized to probe for 2-HG and Glu or GLX have been developed and can be used to monitor the metabolic changes associated with treatment [23,26,32,41,42]. Thus, our observations are also relevant to the clinical setting.

When reviewing the metabolic alterations common to all three glioma models in this study, we found that in every case not only did we detect an early drop in 2-HG, but we also detected an early increase in Glu, GLX and NAA levels. NAA is present in neurons, and its early increase in this in vivo study could reflects an increase in neuronal density, even though differences in tumor volume could not yet be detected at the early time points of treatment. Nonetheless it should be noted that the increase in NAA was not observed in our study at the later time point, and a clinical study monitoring the effect of IDH305 did not observe a significant change in NAA level following treatment, pointing to the need for further investigations to assess the value of NAA as an early biomarker of response [33]. The drop in 2-HG reflects drug delivery and mutant IDH inhibition. It is also in line with reports in both preclinical and clinical studies [26,33,43]. The changes in 2-HG and Glu levels are also in line with our previous observations in the U87IDHmut cells treated with either AG-120 or AG-881 that showed a significant decrease in 2-HG and a significant increase in Glu [34]. GLX, which is also increased in this in vivo study, reflects the sum of Glu and Gln. Those metabolites are in equilibrium but Gln levels in our models were much smaller than those of Glu. Thus, the increase in GLX is in fact a reflection of the increase in Glu, with changes in Gln less consistent across our models, in line with our findings in cells that did not point to a consistent increase in Gln following treatment. More importantly, the increase in GLX level is consistent with the clinical study of IDH305 that reported that the MRI changes indicative of reduced cell density were inversely correlated with 2-HG/GLX [33].

The increase in Glu observed both in cells and in vivo reflects a reversal of metabolic changes previously reported to be associated with the IDH mutation by our group and others in cell models [37,44] and in patient samples [45]. Interestingly however, not all ^1^H-MRS detectable metabolic alterations associated with the IDH1 mutation appear to be reversed with treatment. We have shown that cells genetically engineered to express mutant IDH1 increase 2-HG and also decrease Glu, phosphocholine and lactate [37]. However, our previous study in cells [34], as well as the results from this in vivo study, observe a drop in 2-HG and an increase in Glu following mutant IDH inhibition, but no significant changes in lactate or a consistent increase in choline-containing metabolites. Our findings therefore point to the complexity of the molecular biological and metabolic changes that occur in mutant IDH1 tumors during their development and in response to treatment [46]. Most importantly however, the consistency of our metabolic observations across cell and in vivo studies, their association in cells with reduced clonogenic potential, and in vivo with inhibition of tumor growth and enhanced animal survival, point to the value and reliability of reduced 2-HG and elevated Glu and GLX as noninvasive biomarkers of mutant IDH inhibition.

## 4. Materials and Methods

### 4.1. Cell Culture

U87IDHmut glioblastoma cells engineered to express the IDH1 R132H mutant gene, courtesy of the Phillips Lab (UCSF), were maintained in culture as described [47]. SF10417 patient-derived mutant IDH1 oligodendroglioma, courtesy of the Costello Lab (UCSF), were cultured as described [48]. BT257 patient-derived mutant IDH astrocytoma, courtesy of the Weiss lab (University of Calgary), were cultured as described [49]. Authentication of cell lines by short tandem repeat fingerprinting and routine testing for mycoplasma were performed within 6 months of study.

### 4.2. Mutant IDH Inhibitors

Pan-mutant IDH1/2 inhibitor, AG-881, (MedChemExpress) and pan-mutant IDH1 inhibitor, BAY-1436032 (MedChemExpress), were resuspended by sonication in oral suspending vehicle, Ora-plus, (Perrigo).

### 4.3. Animal Models and Study Design

All studies were performed under UCSF Institutional Animal Care and Use Committee approval (AN184161-01E). 6–9 week old female athymic nu/nu mice (20–25 g; Charles River Laboratories, Wilmington, MA, USA) were intracranially injected with ~3 × 10^5^ U87IDHmut cells. 6–9 week old female SCID mice (19–21 g; Fox Chase SCID mice, Charles River Laboratories, Wilmington, MA, USA) were intracranially injected with ~1 × 10^5^ of either BT257 or SF10417 cells from serial orthotopic xenografting as described [49]. Tumor size was evaluated using MRI. Once tumors reached 2–3 mm in diameter, a baseline set of MR studies was performed (see below). This time point was considered day zero (D0). Mice were then randomized into three treatment groups and treated daily (Monday to Friday) per os (p.o.) with one of the following: (1) 50 mg/kg AG-881 [38]; (2) 150 mg/kg BAY-1436032 [43]; (3) 4 mL/kg Ora-plus (for controls). MR studies were then repeated at regular intervals depending on the tumor model and continued until the animal had to be sacrificed (Figure 1).

### 4.4. In Vivo MR Studies

The ^1^H-MRS data was acquired for each treatment group in each model as follows: U87IDHmut model (controls *n* = 4, AG-881-treated *n* = 4, BAY-1436032-treated *n* = 5); BT257 model (controls *n* = 10, AG-881-treated *n* = 6, BAY-1436032-treated *n* = 5); and SF10417 model (controls *n* = 6, AG-881-treated *n* = 6, BAY-1436032-treated *n* = 5).

MR studies were performed using a vertical 14.1T scanner (Agilent Technologies, Varian Medical Systems, Palo Alto, CA, USA), equipped with a single channel 40 mm ^1^H coil. Tumor volume was evaluated from axial T2-weighted images acquired using a spin-echo multi-slice sequence using the following parameters: TE = 20 ms, TR = 1200 ms, FOV = 30 × 30 mm^2^, matrix 256 × 256, slice thickness 1mm, NA = 2. Tumor volume was determined by summing manually contoured tumor areas in each axial slice and multiplying by slice thickness using in-house MR software [50]. ^1^H-MRS spectra were then acquired at two time points: an early time point prior to when a significant inhibition in tumor growth relative to controls could be detected by MRI, and a late time point after a significant inhibition in tumor growth was detected (except for the U87IDHmut model because, as described in the results section, its rate of tumor growth was not inhibited with treatment prior to the death of all control animals) (Figure 1). Spectra were acquired from a single 8 mm^3^ voxel placed in the center of the tumor region using the PRESS (point-resolved spectroscopy) sequence with: TE = 20 ms, TR = 4000 ms, NA = 512, 10,000 points, spectral width 10,000 Hz. The spectra were analyzed using LCModel (0.6–4.2 ppm) with a basis set that includes 2-hydroxyglutarate (2-HG), 4-aminobutyrate (GABA), alanine (Ala), N-acetylaspartate (NAA), glutamine (Gln), glutamate (Glu), glutathione (GSH), total creatine (sum of creatine and phosphocreatine, tCr), total choline (sum of choline, phosphocholine and glycerophosphocholine, tCho), aspartate (Asp), lactate (Lac), taurine (Tau), glycine (Gly), glucose (Glc), scylloinositol (sI), myoinositol (mI), following normalization to total signal [47,51,52]. The average level of each metabolite, $\bar{C}$, and standard deviation for each metabolite, $\sigma \left(\bar{C}\right)$, were calculated using the concentrations for each animal, Cj, and its associated Cramer Rao Lower Bounds (CRLB) determined by LCModel as follows [52,53]:

$$\bar{C}=\sum \omega_{j}C_{j}/\sum \omega_{j}$$

$$\sigma \left(\bar{C}\right)=1/\sqrt{\sum \omega_{j}}$$

$$\omega_{j}=1/\sigma_{j}^{2}$$

$$\sigma_{j}={\left(\%CRBL\right)}_{j}\times C_{j}/100$$

where *j* corresponds to an individual animal.

### 4.5. Statistical Analysis

Student’s unpaired two-tailed t-test (GraphPad Prism) was used to determine the statistical significance of differences between treatment groups, and *p*-value ≤ 0.05 was considered significant. For the 17 metabolites compared between the treatment groups we also applied a Bonferroni multiple comparisons correction (*p*-value < 0.003 considered significant). Results shown as mean ± standard deviation. Kaplan-Meier survival curves with Log-rank test were used to compare survival.

## 5. Conclusions

In this study, we noninvasively monitored metabolic alterations in response to longitudinal mutant IDH inhibition using high resolution ^1^H-MRS in mutant IDH1 glioma models. In the genetically engineered U87IDHmut model as well as in two patient-derived BT257 and SF10417 mutant IDH1 glioma models, treatments with either AG-881 or BAY-1436032 resulted in a consistent reduction in 2-HG, confirming brain penetration and action of the IDH inhibitors. Moreover, we detected an early and significant increase in Glu and GLX levels prior to any detectable inhibition in tumor growth. Our study thus points to the increase in Glu and GLX together with the drop in 2-HG as potential translatable early metabolic biomarkers of glioma response to mutant IDH inhibition, which, in combination with the monitoring of tumor volume, could serve to enhance the reliability of noninvasive monitoring of response to mutant IDH inhibitors.

## Figures and Tables

**Table 1:** Metabolite levels significantly different between control and treated U87IDHmut tumors at D5 ± 1.

Metabolite	Cramer-Rao Lower Bounds (%)			Concentration (AU)		
	Control	AG-881	BAY-1436032	Control	AG-881	BAY-1436032
2HG	8	22	10	4.10 ± 0.17	1.73 ± 0.12	3.09 ± 0.12
GSH	4	5	4	4.72 ± 0.07	5.12 ± 0.08	4.26 ± 0.06
NAA	7	8	7	3.36 ± 0.13	4.97 ± 0.15	4.62 ± 0.13
Lac	5	7	6	15.04 ± 0.27	-	8.67 ± 0.25
Glc	30	28	22	1.12 ± 0.22	-	2.25 ± 0.12
mI	14	6	7	2.17 ± 0.07	2.97 ± 0.09	2.49 ± 0.07
Asp	7	13	5	6.53 ± 0.27	1.19 ± 0.12	14.35 ± 0.29
Tau	4	4	6	9.30 ± 0.15	8.04 ± 0.19	6.45 ± 0.14
GABA	6	9	7	7.30 ± 0.20	2.57 ± 0.14	3.87 ± 0.14
Ala	13	15	18	3.03 ± 0.10	1.09 ± 0.05	0.81 ± 0.04
Gln	6	21	8	3.15 ± 0.11	1.54 ± 0.09	1.74 ± 0.06
Glu	3	3	2	12.17 ± 0.17	17.35 ± 0.24	16.98 ± 0.17
Gly	7	13	7	7.20 ± 0.21	0.30 ± 0.04	-
sI	44	13	17	0.15 ± 0.03	-	-
tCho	1	1	1	9.78 ± 0.05	14.51 ± 0.07	10.31 ± 0.05
tCr	7	12	8	2.03 ± 0.06	1.53 ± 0.07	1.36 ± 0.05
GLX	3	3	2	16.47 ± 0.22	18.74 ± 0.25	19.08 ± 0.20

**Table 2:** Metabolite levels significantly different between control and treated BT257 tumors at early (D7 ± 2) and late (D15 ± 1) time points.

D7 ± 2						
Metabolite	Cramer-Rao Lower Bounds (%)			Concentration (AU)		
	Control	AG-881	BAY-1436032	Control	AG-881	BAY-1436032
2HG	6	7	5	8.63 ± 0.16	5.57 ± 0.13	6.89 ± 0.13
GSH	6	6	5	2.19 ± 0.04	2.34 ± 0.05	2.53 ± 0.05
NAA	4	4	3	8.54 ± 0.10	11.38 ± 0.10	13.44 ± 0.11
Lac	14	8	6	5.73 ± 0.22	4.48 ± 0.10	4.29 ± 0.11
mI	4	3	2	19.83 ± 0.14	15.27 ± 0.12	20.38 ± 0.13
Asp	22	28	7	6.30 ± 0.19	2.26 ± 0.13	7.75 ± 0.23
Tau	5	3	3	10.74 ± 0.13	14.16 ± 0.16	10.42 ± 0.13
GABA	5	12	4	12.64 ± 0.17	6.62 ± 0.14	13.28 ± 0.19
Ala	22	10	10	0.98 ± 0.05	1.66 ± 0.07	-
Gln	6	5	5	4.93 ± 0.08	4.55 ± 0.09	4.48 ± 0.08
Glu	7	4	4	7.59 ± 0.14	11.10 ± 0.14	11.22 ± 0.16
Gly	5	4	4	27.39 ± 0.35	21.03 ± 0.19	16.23 ± 0.15
sI	14	8	13	0.46 ± 0.02	0.66 ± 0.02	0.88 ± 0.03
tCho	1	1	1	12.70 ± 0.04	8.70 ± 0.04	9.31 ± 0.04
tCr	4	2	2	7.87 ± 0.06	12.13 ± 0.09	8.75 ± 0.07
GLX	5	4	3	13.19 ± 0.18	15.21 ± 0.20	16.39 ± 0.19
**D15 ± 1**						
2HG	4	7	4	14.79 ± 0.20	4.73 ± 0.17	7.42 ± 0.17
GSH	11	7	4	3.41 ± 0.06	2.20 ± 0.07	1.89 ± 0.04
NAA	4	4	4	11.45 ± 0.10	-	8.73 ± 0.08
Lac	8	13	10	7.28 ± 0.19	5.94 ± 0.38	3.18 ± 0.17
mI	3	2	2	19.45 ± 0.11	22.92 ± 0.25	22.49 ± 0.17
Asp	9	10	6	8.44 ± 0.31	4.75 ± 0.24	6.49 ± 0.18
Tau	4	5	4	11.04 ± 0.12	15.38 ± 0.38	11.68 ± 0.17
GABA	4	6	5	20.74 ± 0.18	13.82 ± 0.37	8.48 ± 0.14
Ala	10	11	15	2.29 ± 0.06	-	1.00 ± 0.03
Gln	7	6	6	5.47 ± 0.10	3.90 ± 0.12	2.48 ± 0.07
Glu	5	7	4	7.12 ± 0.13	9.89 ± 0.31	10.83 ± 0.15
Gly	4	6	4	19.87 ± 0.15	13.32 ± 0.38	19.19 ± 0.17
sI	13	14	11	0.57 ± 0.03	0.81 ± 0.06	0.34 ± 0.02
tCho	1	1	1	12.37 ± 0.05	10.60 ± 0.06	11.45 ± 0.06
tCr	3	3	3	7.89 ± 0.06	10.79 ± 0.16	13.31 ± 0.11
GLX	4	5	4	12.03 ± 0.18	13.90 ± 0.34	14.91 ± 0.19

**Table 3:** Metabolite levels significantly different between control and treated SF10417 tumors at early (D6 ± 2) and late (D14 ± 2) time points.

D6 ± 2						
Metabolite	Cramer-Rao Lower Bounds (%)			Concentration (AU)		
	Control	AG-881	BAY-1436032	Control	AG-881	BAY-1436032
2-HG	4	5	5	7.73 ± 0.13	7.17 ± 0.11	5.98 ± 0.11
GSH	5	5	3	2.71 ± 0.05	1.73 ± 0.03	3.08 ± 0.04
NAA	4	3	3	6.37 ± 0.10	8.10 ± 0.06	8.57 ± 0.06
Lac	30	13	7	3.62 ± 0.19	-	3.13 ± 0.06
Glc	29	14	5	3.17 ± 0.20	3.98 ± 0.12	5.39 ± 0.12
mI	3	2	2	8.99 ± 0.07	7.00 ± 0.07	8.13 ± 0.04
Asp	6	5	5	11.37 ± 0.26	-	5.34 ± 0.12
Tau	5	4	3	5.27 ± 0.10	8.66 ± 0.08	13.07 ± 0.09
GABA	7	4	5	4.34 ± 0.11	6.75 ± 0.09	5.93 ± 0.09
Ala	36	11	10	0.47 ± 0.04	-	0.83 ± 0.03
Gln	16	6	4	1.71 ± 0.09	3.28 ± 0.07	2.67 ± 0.05
Glu	6	5	3	4.93 ± 0.10	5.92 ± 0.08	11.24 ± 0.09
Gly	5	3	5	12.68 ± 0.16	13.79 ± 0.11	10.25 ± 0.10
sI	30	13	10	0.29 ± 0.02	0.38 ± 0.03	0.18 ± 0.01
tCho	1	1	1	9.75 ± 0.04	7.99 ± 0.03	6.18 ± 0.03
tCr	3	2	2	9.77 ± 0.07	7.20 ± 0.05	8.03 ± 0.04
GLX	5	4	3	7.31 ± 0.15	11.48 ± 0.11	13.54 ± 0.11
**D14 ± 2**						
2-HG	3	4	4	9.91 ± 0.16	6.56 ± 0.13	8.09 ± 0.12
GSH	5	5	5	1.71 ± 0.05	3.31 ± 0.06	2.62 ± 0.04
NAA	4	3	3	9.42 ± 0.17	6.01 ± 0.10	9.04 ± 0.08
Lac	7	15	7	2.43 ± 0.17	-	-
Glc	10	8	12	3.19 ± 0.18	-	-
mI	2	3	3	10.21 ± 0.09	9.72 ± 0.09	8.34 ± 0.07
Asp	6	7	7	15.89 ± 0.42	9.05 ± 0.32	5.50 ± 0.14
Tau	4	3	4	5.02 ± 0.12	7.46 ± 0.12	13.98 ± 0.13
GABA	8	5	5	4.71 ± 0.15	-	7.71 ± 0.12
Ala	16	42	17	0.96 ± 0.15	-	-
Gln	25	9	5	0.88 ± 0.09	1.74 ± 0.10	3.20 ± 0.06
Glu	6	3	6	5.26 ± 0.14	6.93 ± 0.10	7.63 ± 0.12
Gly	4	4	5	7.88 ± 0.19	14.64 ± 0.14	10.58 ± 0.17
sI	20	19	16	0.22 ± 0.03	-	0.32 ± 0.01
tCho	1	1	1	9.28 ± 0.05	8.89 ± 0.05	6.06 ± 0.03
tCr	3	2	2	7.41 ± 0.11	10.62 ± 0.10	-
GLX	6	3	4	5.98 ± 0.18	7.90 ± 0.12	11.69 ± 0.15

## Data Availability

The data presented in this study are openly available in Dryad at 10.7272/Q6B856C7.
